# Supplementary material for: ER tethering and active transport govern condensate diffusion during hyperosmotic stress
Source: bioRxiv. 2025 Jun 17:2025.06.13.659610. Preprint. [Version 1] doi: 10.1101/2025.06.13.659610 (PMC12262303; doi:10.1101/2025.06.13.659610)
Supplement: Supplement 1 [file media-1.pdf]

Supplementary Information

ER tethering and active transport govern condensate diffusion during hyperosmotic stress

Bisal Halder[1,2#], Guoming Gao[1,3#], Armin Ahnoud[1], Shelby Stakenas[1,2], Emily R. Sumrall[1] and Nils G. Walter[1,2*]

[1]Center for RNA Biomedicine, University of Michigan, Ann Arbor, MI 48109, USA

[2]Department of Chemistry, University of Michigan, Ann Arbor, MI 48109, USA

[3]Current Address: Division of Biology and Biological Engineering, California Institute of Technology, Pasadena, CA 91125, USA

[#]These authors contributed equally to this work

*Correspondence: nwalter@umich.edu

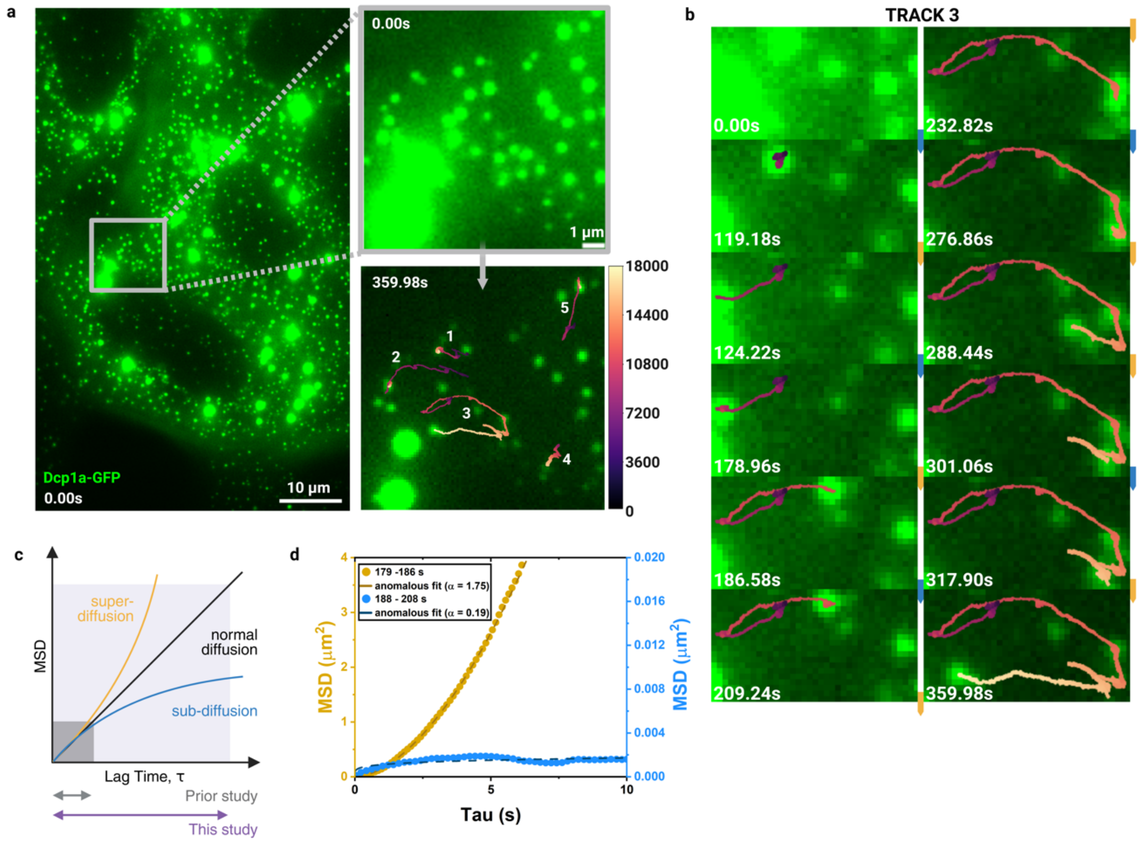

Fig. S1 | Dual-mode dynamics of hyperosmotic phase separation (HOPS) condensates reproduced under 50 Hz imaging frequency

a | Representative images of U2OS cells expressing eGFP-labeled DCP1A under hyperosmotic stress conditions. Enlarged views emphasize trajectories of hyperosmotic stress-induced HOPS condensates, where individual particle locations throughout a trajectory are colored by frame number. **b |** Time-lapse zoomed-in images of a representative HOPS condensate trajectory (#3 shown in **a**), showing both predominant sub diffusion and the occasional super diffusion. **c |** The mean squared displacement (MSD) as a function of lag time (τ) demonstrates the ability of extended time scales to differentiate between various diffusion modes. **d |** Comparison of MSD-τ curves between the sub-diffusive portions and the super diffusion portions of the same set of HOPS condensate trajectories.

Table S1 | Theoretical and experimentally determined diffusion coefficient of HOPS condensate and GEM particles from four representative particles each

	Radius (nm)	Theoretical D ($\mu m^2/s$)	Experimental D ($\mu m^2/s$)
HOPS Particle #1	80.84	0.0394	0.000232
HOPS Particle #2	186.70	0.0170	0.000189
HOPS Particle #3	272.16	0.0117	0.000319
HOPS Particle #4	313.11	0.0101	0.000118
GEM Particle #1	40	0.0797	0.136530
GEM Particle #2	40	0.0797	0.017967
GEM Particle #3	40	0.0797	0.022489
GEM Particle #4	40	0.0797	0.014519
